# Impact of the Covid-19 epidemic and related social distancing regulations on social contact and SARS-CoV-2 transmission potential in rural South Africa: analysis of repeated cross-sectional surveys

**DOI:** 10.1186/s12879-021-06604-8

**Published:** 2021-09-08

**Authors:** Nicky McCreesh, Vuyiswa Dlamini, Anita Edwards, Stephen Olivier, Njabulo Dayi, Keabetswe Dikgale, Siyabonga Nxumalo, Jaco Dreyer, Kathy Baisley, Mark J. Siedner, Richard G. White, Kobus Herbst, Alison D. Grant, Guy Harling

**Affiliations:** 1 Department of Infectious Disease Epidemiology, London School of Hygiene and Tropical Medicine, London, UK; 2 Africa Health Research Institute, KwaZulu-Natal, Durban, South Africa; 3 Harvard Medical School and the Medical Practice Evaluation Center, Massachusetts General Hospital, Boston, MA USA; 4 DSI-MRC South African Population Research Infrastructure Network, Durban, South Africa; 5 TB Centre, London School of Hygiene and Tropical Medicine, London, UK; 6 School of Laboratory and Medical Sciences, College of Health Sciences, University of KwaZulu-Natal, KwaZulu-Natal, Durban, South Africa; 7 School of Public Health, University of the Witwatersrand, Johannesburg, South Africa; 8 Institute for Global Health, University College London, London, UK; 9 Department of Epidemiology & Harvard Center for Population and Development Studies, Harvard T.H. Chan School of Public Health, Boston, MA USA; 10 MRC/Wits Rural Public Health and Health Transitions Research Unit (Agincourt), School of Public Health, University of the Witwatersrand, Johannesburg, South Africa

**Keywords:** COVID-19, Contact survey, Reproduction number, Social contacts, Indoor

## Abstract

**Background:**

South Africa implemented rapid and strict physical distancing regulations to minimize SARS-CoV-2 epidemic spread. Evidence on the impact of such measures on interpersonal contact in rural and lower-income settings is limited.

**Methods:**

We compared population-representative social contact surveys conducted in the same rural KwaZulu-Natal location once in 2019 and twice in mid-2020. Respondents reported characteristics of physical and conversational (‘close interaction’) contacts over 24 hours. We built age-mixing matrices and estimated the proportional change in the SARS-CoV-2 reproduction number (R_0_). Respondents also reported counts of others present at locations visited and transport used, from which we evaluated change in potential exposure to airborne infection due to shared indoor space (‘shared air’).

**Results:**

Respondents in March–December 2019 (n = 1704) reported a mean of 7.4 close interaction contacts and 196 shared air person-hours beyond their homes. Respondents in June-July 2020 (n = 216), as the epidemic peaked locally, reported 4.1 close interaction contacts and 21 shared air person-hours outside their home, with significant declines in others’ homes and public spaces. Adults aged over 50 had fewer close contacts with others over 50, but little change in contact with 15–29 year olds, reflecting ongoing contact within multigenerational households. We estimate potential R_0_ fell by 42% (95% plausible range 14–59%) between 2019 and June-July 2020.

**Conclusions:**

Extra-household social contact fell substantially following imposition of Covid-19 distancing regulations in rural South Africa. Ongoing contact within intergenerational households highlighted a potential limitation of social distancing measures in protecting older adults.

**Supplementary Information:**

The online version contains supplementary material available at 10.1186/s12879-021-06604-8.

## Background

The rapid spread of SARS-CoV-2 in 2020 has harmed populations both directly through Covid-19 morbidity and mortality, and indirectly via both less support for other health conditions [1, 2] and economic impacts arising from government-imposed and self-directed reductions in social interaction [3, 4]. Local physical distancing regulations including mandatory ‘stay at home’ orders, restrictions on public gatherings, and banning of inter-household contact have been common during the pandemic [5]. Such non-pharmaceutical interventions (NPI) were variably implemented and often enforced more stringently in lower-income settings in sub-Saharan Africa [6]. Understanding the impact of both NPIs and personal decisions is vital to determining trade-offs between epidemic control and non-Covid wellbeing.

The impact of NPIs is likely to vary substantially across countries, reflecting differences in both demographic composition and social dynamics. There is particular concern that official movement limitations may have limited impact in settings where informal work is common and economic safety nets are limited [7, 8]. These concerns will be particularly important if the global pandemic follows the example of past infectious diseases and has its greatest impact on marginalized and previously disadvantaged populations [9, 10].

Quantitative data on relevant interpersonal interaction are central to such assessments, both directly for planning locally relevant evidence-based responses and for parameterisation of mathematical models of SARS-CoV-2 transmission and control policies. Movement data from sources linked to smartphones can indicate likely changes in contact patterns, they do not account for the detailed, non-random social interaction that often typifies human behaviour [11], and in settings with low smartphone penetration, reliance on such data can lead to biased results.

Detailed quantitative social contact surveys have been conducted during the Covid-19 pandemic, primarily in higher-income settings. These include online surveys using de novo convenience recruitment in Europe [12, 13], and existing online panels in Europe [14, 15] and the United States [16]. Telephonic surveys have been conducted using random digit dialling in China [17], and existing cohorts in Kenya [18].

Maximizing the benefit of these Covid-19 social contact studies requires careful study design. First, a clear sampling frame rather than a convenience sample allows stronger inference to a source population. Second, having comparable pre-pandemic data allows a clear measure of change to be assessed—there is danger of recall bias if questions are asked retrospectively about pre-pandemic days, and of secular change prior to Covid-19 if using previously collected data from too long ago. Third, longitudinal data within the epidemic’s progress allows judgement of the effects of changing policy and compliance willingness. Fourth, given evidence for aerosolized SARS-CoV-2 transmission [19], adding information on contact-time occurring in indoor congregate settings and transport can broaden our understanding of risk.

South Africa implemented an early, stringent national lockdown in March 2020, which may have initially delayed the national epidemic [20]; however regulations were relaxed from May onwards and case numbers increased rapidly, peaking in July before falling back. In this paper, we compare data from two studies conducted using comparable study instruments in 2019 and 2020, both using samples drawn from the same census sampling frame in rural South Africa. The 2020 data include two rounds of data collection, covering the first wave of Covid-19 in the local area. We use these data to estimate the reduction in potential reproduction number of SARS-CoV-2 between surveys, and to determine where contact beyond the home continued during lockdown periods.

## Methods

We used data from two surveys conducted in the southern section of the Africa Health Research Institute (AHRI) demographic surveillance area in 2019 and 2020 [21]. AHRI maintains an active thrice-yearly census of all households ~ 21,000 households in this area of ~ 850 km^2^ in rural uMkhanyakude district, KwaZulu-Natal province, including one small town [22]. uMkhanyakude ranks among the most deprived districts nationally in terms of health and socioeconomic status.

The 2019 data were collected as part of Umoya omuhle (UO), a programme exploring novel approaches to prevention of drug-resistant *Mtb* transmission in health facilities [23]. UO sampled 3093 census adults (aged 18 and above) residing within the census surveillance area and the catchment area of two primary care centres (one in town, one rural). Sampling was random, stratified by residential area (~ 350 households per area) and with probability proportional to the number of eligible people in each area, based on the most recent census conducted prior to area entry. Data collection was conducted March to December 2019 at respondents’ homes.

**Fig. 1:**
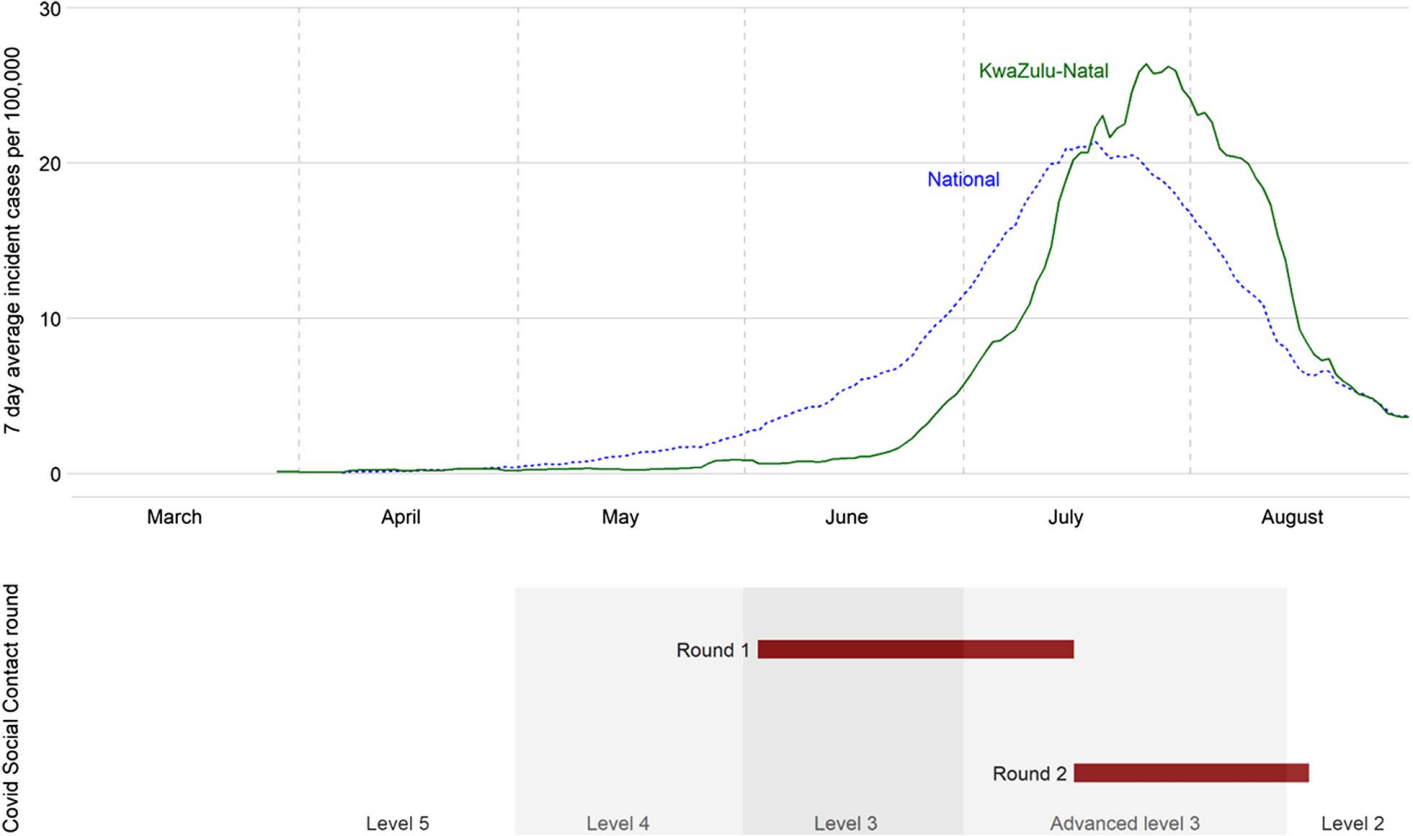
South African smoothed Covid-19 case incidence rate, government lockdown and 2020 survey dates in 2020. Levels refer to national non-pharmaceutical interventions (“lockdown”) with strictness declining from level 5 over time (details on interventions at each level in Additional file 1: Material S2). Incidence rates are 7-day running mean values computed from case data collated at [26]

The 2020 data were collected as part of a longitudinal Covid-19 surveillance project [24]. The Covid Social Contact (CSC) sub-study used an age/sex stratified sample of one person aged 15 and above from each of 400 census households. Inclusion criteria included participation in Vuk’uzazi, a recent population-wide chronic health screening study [25], allowing intentional oversampling of individuals with locally prevalent health conditions (tuberculosis, hypertension, diabetes, obesity, COPD or asthma). Contact was made telephonically based on previously provided numbers. We analysed two rounds of data collected between 3 June and 16 July (2020 R1), and 16 July and 17 August (2020 R2), as Covid-19 peaked in KwaZulu-Natal (Fig. 1).

Both studies used structured electronic REDCap interviews (Additional file 1: Material S1). After confirming socio-demographic characteristics, respondents were asked to report on three forms of in-person social interaction. First, they were asked to list all indoor locations visited over a 24 h period. UO asked about a randomly selected day in the past week; CSC asked about the day prior to interview, limiting data largely to Sunday to Thursday. Follow up questions covered the type of location, length of time spent there and the number of people present. Second, respondents were asked who they had directly interacted with over the 24 h period – involving either physical contact or a minimum-three-word conversation (‘close interaction contact’). Respondents were then for each such close interaction contact asked to report their ages and sexes and the duration of time spent together; UO asked these follow-up questions about a random 10 contacts, or all contacts if 10 or fewer were reported while CSC asked about all contacts. Third, respondents were asked about any transport used over the past day, and then how long any trips took and many people shared the transport with them. CSC only asked buildings and transport questions to a random half of sampled individuals per round.

## Statistical analyses

We grouped respondents and their close interaction contacts by age into four categories (15–29, 30–49, 50–64 and 65 +) and described respondent characteristics for each survey round. All subsequent analyses weighted the data for sampling and non-response to match the census population and to make methods comparable across surveys (further details in Additional file 1: Material S3). We first calculated the mean number of close interaction contacts per person per day by respondent characteristics (sex, age, household size) and by whether the contact was a household member. We then built social contact age mixing matrices for each round, adjusting our raw results to ensure that the matrices were symmetric using the census population age structure. From these values we calculated the change between 2019 and each 2020 round.

In the absence of a robust estimate of the basic reproduction number (R_0_) for SARS-CoV-2 in rural South Africa without social distancing (i.e., with 2019 social contact patterns), we estimated the relative reduction in R_0_ between rounds, assuming the per-contact transmission probability remained constant. (Actual reductions will have been greater to the extent that face coverage increased in 2020 but we cannot precisely estimate either the degree to which this occurred, especially within households, or the degree of protection conferred.) For our estimated reduction we used the next generation matrix of the age-contact matrix, defining R_0_ as the dominant eigenvalue [27]. To assess uncertainty, we generated mean and 95% plausible intervals using 10,000 independent bootstrapped samples of each survey, and calculated the relative reduction in each pair of samples. The bootstrapped samples were generated by re-sampling respondents with replacement within age categories, and re-sampling contacts with replacement from the set of all contacts of the respondent [28].

Finally, we calculated the change in potential exposure to airborne infection using location and transport data by calculating for each respondent per day: the proportion who visited any location/transport type; mean hours spent in the location if visited; mean people present per visit; mean ‘shared air person-hours’ if visited; and finally mean shared air person-hours across all respondents. For this analysis we merged the two CSC rounds since each respondent provided one datapoint. We tested for significant differences in each measure using logistic or linear bivariate regression including indicator variables for study round.

We conducted several sensitivity analyses: (i) including only data on Sundays to Thursdays, giving each day equal weight, to compare only same-day data across the two surveys; (ii) excluding respondents with any missing close interaction contact age data; iii) excluding close interaction contacts of less than 15 min duration, as the probability of transmission is lower for shorter contact durations [29, 30]; iv) incorporating children into our analysis, using UO and CSC data on adult-reported contact with children, and past South African data about child-child contacts; (v) including only individuals aged ≥ 18, to make the two datasets comparable on age range; (vi) limiting UO data to the period June–August 2019 to ensure seasonal comparability; (vii) weighted the 2019 data to the full census population based on urbanicity (further details in Additional file 1: Material S3).

Ethical approval for UO was granted by the Biomedical Research Ethics Committee (REC) of the University of KwaZulu-Natal (UKZN) (BE662/17) and the London School of Hygiene & Tropical Medicine (14,640); ethical approval for CSC was granted by UKZN BREC (BE290/16) and University College London REC (15231/013). Informed consent for participation was recorded in writing for UO and telephonically for CSC.

## Results

Of the 3093 people sampled for UO, 1723 (56%) were successfully contacted, 299 (10%) were dead or reported to have out-migrated, and 1071 (35%) could not be contacted. Of those successfully contacted, 1704 (99%) completed an interview. Of the 400 individuals sampled for CSC, 27 (7%) were dead or had out-migrated, and 102 (26%) could not be contacted. Of those successfully contacted, 216 (80%) completed an interview. At R2 follow-up, 202 of the 216 (94%) completed a second interview and eight previously uncontactable individuals were reached for a first interview. The raw age-sex structures of UO and CSC differed from one-another by design (Table 1).

**Table 1:** Descriptive statistics of respondents to the Umoya omuhle (2019) and Covid Social Contacts (2020) studies

	2019		2020 R1: June-July				2020 R2: July–August					
	All		Close contact		Buildings & transport		Close contact		Buildings & transport			Census population
Age												
15–29	613	36%	36	17%	15	14%	32	15%	17		17%	42%
30–49	535	31%	50	23%	28	26%	47	22%	23		23%	33%
50–64	342	20%	64	30%	35	33%	65	31%	28		28%	16%
65 +	214	13%	66	31%	29	27%	66	31%	33		33%	10%
Sex												
Male	751	44%	101	47%	49	46%	97	46%	48		48%	42%
Female	953	56%	115	53%	58	54%	113	54%	53		52%	58%
Household size												
1–3	293	17%	61	28%	33	31%	62	30%	27		27%	19%
4–6	426	25%	60	28%	28	26%	60	29%	29		29%	34%
7–9	429	25%	54	25%	29	27%	48	23%	22		22%	25%
10 +	556	33%	41	19%	17	16%	40	19%	23		23%	22%
Residence												
Urban/peri-urban	837	49%	85	39%	37	35%	84	40%	41		41%	32%
Rural	867	51%	131	61%	70	65%	125	60%	59		58%	59%
Unknown	0		0		0		1	0.5%	1		1.0%	0.4%
Day reported												
Monday	239	14%	49	23%	22	21%	25	12%	15		15%	
Tuesday	242	14%	52	24%	24	22%	26	12%	12		12%	
Wednesday	239	14%	37	17%	20	19%	73	35%	35		35%	
Thursday	251	15%	40	19%	24	22%	70	33%	33		33%	
Friday	261	15%	0		0		1	0%	1		1%	
Saturday	245	14%	0		0		0		0			
Sunday	227	13%	38	18%	17	16%	15	7%	5		5%	
Total	1704		216		107		210		101			36 311

Values are counts and percentages. Census population refers to proportions of residents aged 15 + in the area

The mean number of close interaction contacts varied little by respondent age, sex or household size within rounds, although numbers were lower in the highest age group, and were positively associated with household size (Table 2). Respondents reported a mean of 7.4 close interaction contacts in 2019 (95%CI: 7.1–7.7), 4.1 (95%CI: 3.5–4.6) in 2020 R1 and 4.3 (95%CI: 3.8–4.8) in 2020 R2. Contact reductions were larger for non-household than household member contacts in both absolute and relative terms. Non-household contacts fell from 2.8 (95%CI 2.6–3.1) in 2019 to 0.7 (95%CI 0.4–1.1) in 2020 R1 and 0.5 (95% CI 0.3–0.7) in 2020 R2. Household contacts fell from 4.6 (95% CI 4.5–4.8) in 2019 to 3.4 (95% CI 2.9–3.8) in 2020 R1 and 3.8 (95% CI 3.3–4.3) in 2020 R2.

**Table 2:** Mean close contact numbers by respondent characteristics

	2019		2020 R1		*p*-value	2020 R2		*p*-value
*All contacts*	7.4	[7.1–7.7]	4.2	[3.5–5.0]	< 0.001	4.3	[3.3–5.8]	< 0.001
Age								
15–29	8.1	[7.6–8.6]	4.7	[3.3–6.8]	< 0.001	4.6	[3.5–5.9]	< 0.001
30–49	7.0	[6.7–7.4]	3.6	[2.9–4.4]	< 0.001	4.0	[3.2–5.1]	< 0.001
50–64	7.2	[6.5–8.0]	4.8	[3.7–6.2]	0.001	4.8	[3.7–6.2]	0.001
65 +	6.1	[5.5–6.7]	3.3	[2.7–4.0]	< 0.001	3.4	[2.7–4.2]	< 0.001
Sex								
Male	7.1	[6.7–7.5]	4.6	[3.3–6.3]	0.001	3.4	[2.6–4.5]	< 0.001
Female	7.6	[7.3–8.0]	4.0	[3.3–4.7]	< 0.001	4.9	[4.2–5.6]	< 0.001
Household size								
1–3	6.1	[5.4–6.9]	2.6	[2.0–3.3]	< 0.001	2.2	[1.4–3.4]	< 0.001
4–6	6.9	[6.4–7.4]	4.0	[3.4–4.8]	< 0.001	4.0	[3.3–4.8]	< 0.001
7–9	7.6	[7.1–8.2]	4.2	[3.4–5.1]	< 0.001	4.7	[3.9–5.7]	< 0.001
10 +	8.3	[7.9–8.8]	5.8	[3.6–9.2]	0.054	6.0	[4.6–7.7]	0.002
Residence								
Urban/peri-urban	7.1	[6.7–7.5]	4.5	[3.6–5.7]	< 0.001	4.4	[3.3–5.8]	< 0.001
Rural	7.7	[7.3–8.1]	4.0	[3.1–5.2]	< 0.001	4.4	[3.8–5.0]	< 0.001
**All household members**	4.6	[4.5–4.8]	3.4	[3.0–3.9]	< 0.001	3.8	[3.3–4.4]	0.01
Age								
15–29	4.8	[4.6–5.1]	3.6	[2.7–4.8]	0.018	4.1	[3.0–5.4]	0.19
30–49	4.4	[4.2–4.7]	2.9	[2.3–3.6]	< 0.001	3.6	[2.7–4.6]	0.066
50–64	4.5	[4.2–4.9]	4.2	[3.3–5.5]	0.58	4.0	[3.2–5.1]	0.032
65 +	4.6	[4.1–5.1]	3.0	[2.5–3.7]	< 0.001	3.1	[2.5–3.9]	0.001
Sex								
Male	3.9	[3.7–4.1]	3.3	[2.7–4.1]	0.090	2.7	[2.0–3.6]	0.002
Female	5.1	[4.9–5.4]	3.5	[2.9–4.2]	< 0.001	4.5	[3.9–5.2]	0.073
Household size								
1–3	3.2	[2.9–3.5]	1.6	[1.2–2.2]	< 0.001	1.5	[1.0–2.4]	< 0.001
4–6	3.9	[3.7–4.2]	3.4	[2.9–3.9]	0.025	3.3	[2.7–4.2]	0.11
7–9	4.8	[4.6–5.1]	3.8	[3.1–4.6]	0.012	4.5	[3.7–5.5]	0.50
10 +	5.7	[5.4–6.1]	4.5	[3.1–6.6]	0.15	5.3	[4.1–6.8]	0.52
Residence								
Urban/peri-urban	4.3	[4.1–4.6]	3.8	[2.9–4.9]	0.298	3.7	[2.7–5.0]	0.26
Rural	5.0	[4.8–5.3]	3.3	[2.8–3.9]	< 0.001	4.0	[3.4–4.7]	< 0.001
**All non-household members**	2.8	[2.6–3.1]	0.78	[0.44–1.4]	< 0.001	0.52	[0.52–0.32]	< 0.001
Age								
15–29	3.3	[2.9–3.7]	1.2	[0.42–3.2]	< 0.001	0.52	[0.52–0.19]	< 0.001
30–49	2.7	[2.4–3.0]	0.69	[0.40–1.2]	< 0.001	0.48	[0.48–0.20]	< 0.001
50–64	2.7	[2.0–3.5]	0.53	[0.24–1.1]	< 0.001	0.76	[0.76–0.39]	< 0.001
65 +	1.6	[1.2–2.1]	0.28	[0.14–0.54]	< 0.001	0.26	[0.26–0.10]	< 0.001
Sex								
Male	3.3	[2.9–3.7]	1.3	[0.53–3.1]	< 0.001	0.79	[0.79–0.41]	< 0.001
Female	2.5	[2.2–2.9]	0.48	[0.31–0.75]	< 0.001	0.36	[0.36–0.18]	< 0.001
Household size								
1–3	3.0	[2.4–3.8]	1.0	[0.59–1.6]	< 0.001	0.62	[0.62–0.25]	< 0.001
4–6	3.0	[2.5–3.5]	0.66	[0.38–1.2]	< 0.001	0.68	[0.68–0.32]	< 0.001
7–9	2.8	[2.4–3.4]	0.37	[0.16–0.85]	< 0.001	0.20	[0.20–0.06]	< 0.001
10 +	2.6	[2.3–3.0]	1.3	[0.32–5.1]	0.13	0.67	[0.67–0.23]	< 0.001
Residence								
Urban/peri-urban	2.8	[2.5–3.1]	0.73	[0.45–1.2]	< 0.001	0.71	[0.71–0.38]	< 0.001
Rural	2.7	[2.4–3.0]	0.70	[0.24–2.1]	< 0.001	0.39	[0.39–0.16]	< 0.001

Values are means with 95% confidence intervals in brackets. *p*-values are for comparisons within rows vs. 2019 data

**Fig. 2:**
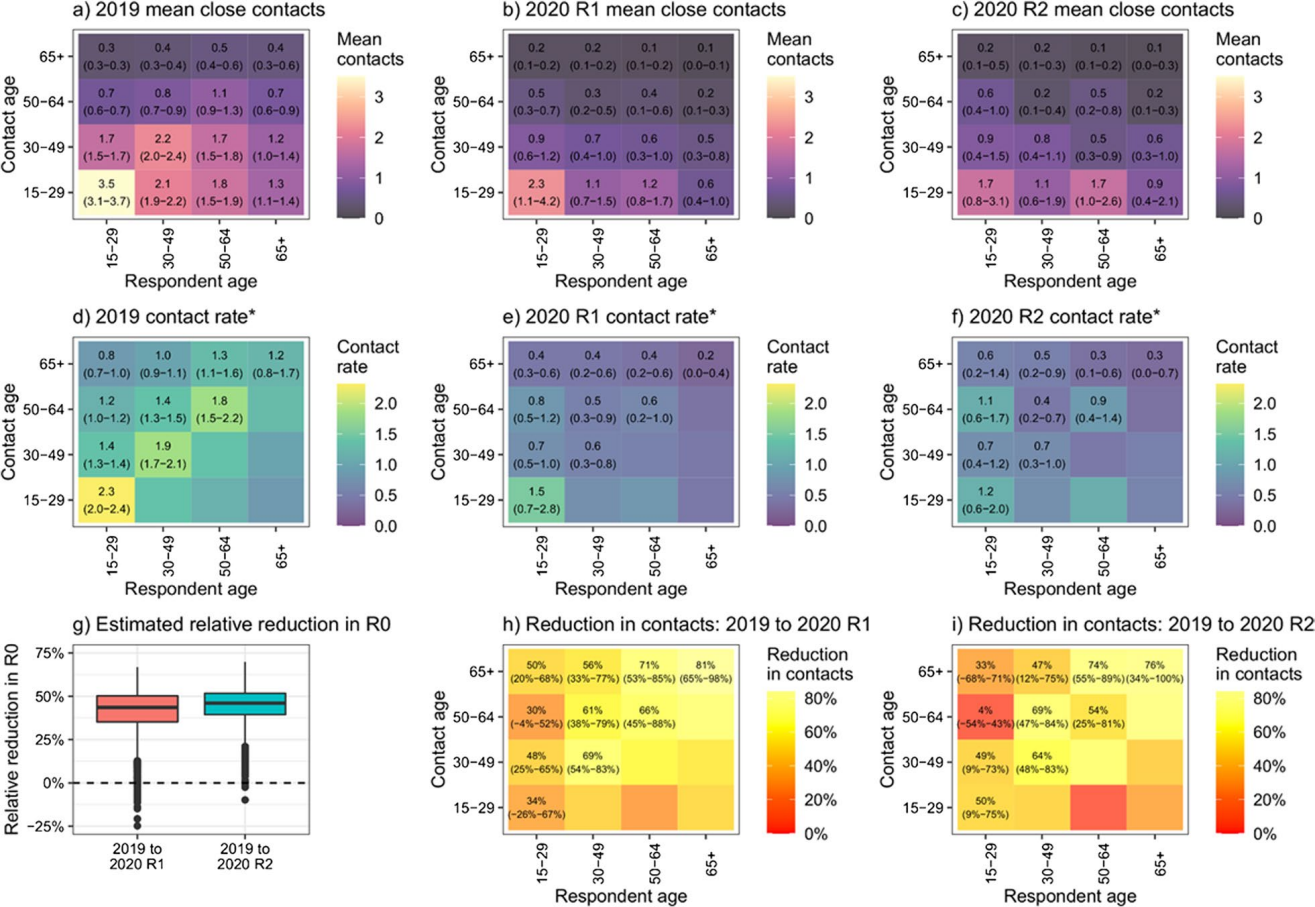
Age-stratified mean (95% plausible range) number of close contacts from survey respondents. Graphs **a**–**c** show the mean number of contacts respondents in each age group reported, by contact age group. Graphs **d**–**f** show the mean rate of contact between respondents in each age group and each other person in the target population, by contact age group. Graph g shows the overall estimated relative reduction in R_0_ from 2019 to 2020. Graphs h and I show the estimated proportional reductions in contact between age groups from 2019 to 2020. *Numbers shown are rates × 10^5^

In 2019 weighted age-mixing matrices, older individuals had fewer close interaction contacts than younger ones, and contact rates for each age group under 65 were highest within the same age group. By June/July 2020 (2020 R1), contact rates were lower for all age combinations, in most cases statistically significantly. The drop was greatest for contact between those aged 65 + , and smallest for contact between those aged 15–29 and 50–64. The drops in contacts adults reported having with children were lower than for between adults, and in most adult age groups the data were consistent with no change in contact rates between adults and children (Additional file 1: Material S4, panel 4). Contact patterns by age changed little between 2020 R1 and R2. The estimated reduction in R_0_ between 2019 and 2020 R1 was 45% (95% plausible range 14–59%), and between 2019 and 2020 R2 was 45% (95% plausible range 24–61%) (Fig. 2, a-c).

Sharing of indoor space more generally also fell between 2019 and mid-2020: mean shared air person-time beyond respondents’ own homes (including transport) fell from 196 to 21 person-contact hours (Table 3). Mean time spent at one’s own home rose by almost three hours per day, from an average of 19 to 22 hours, although there was no significant change in person-contact hours. All other location types except public transport saw substantially reduced overall person-contact hours, largely due to fewer people present in the location, rather than changes in the time spent per visit. The proportion of people reporting clinic visits almost doubled, from 2.3% to 4.3%, although the change was not significant (p = 0.20). The proportion of respondents who reported visiting other people’s households and ‘other’ locations fell, from 27 to 6%, and 32% to 24%, respectively.

**Table 3:** Location and transport patterns of respondents in 2019 and 2020

Location		Proportion visited (%)		*p*-value	Hours if visited		*p*-value	People per visit		*p*-value	Contact-hours if visited		*p*-value	Contact-hours		*p*-value
Own home	2019	98.6	[97.9, 99.1]		19.0	[18.7, 19.2]		5.34	[5.18, 5.50]		107	[103, 111]		106	[102, 110]	
	2020	96.2	[88.7, 98.8]	0.11	21.9	[20.9, 22.9]	< 0.001	5.63	[4.96, 6.39]	0.43	121	[104, 140]	0.14	116	[99.6, 136]	0.26
Other house	2019	26.9	[24.8, 29.1]		6.2	[5.72, 6.77]		10.7	[6.90, 16.6]		71.3	[48.4, 105]		19.1	[12.9, 28.4]	
	2020	6.26	[2.89, 13.9]	< 0.001	12.8	[6.15, 26.7]	0.11	2.67	[2.02, 3.53]	< 0.001	33.1	[16.8, 65.2]	0.027	2.07	[0.63, 6.81]	< 0.001
Clinic	2019	2.27	[1.65, 3.12]		5.08	[3.76, 6.85]		135	[67.6, 269]		561	[258, 1219]		12.8	[5.72, 28.4]	
	2020	4.27	[1.76, 10.0]	0.19	4.88	[3.27, 7.27]	0.86	26.5	[15.1, 46.6]	0.025	150	[78.5, 285]	0.069	6.4	[2.69, 15.1]	0.28
Other locations ^†^	2019	32.3	[30.1, 34.6]		6.09	[5.78, 6.42]		92.4	[76.8, 111]		492	[392, 618]		159	[125, 202]	
	2020	24.5	[17, 33.9]	0.11	5.38	[3.77, 7.68]	0.46	16.0	[7.53, 34.0]	< 0.001	39.0	[24.6, 61.8]	< 0.001	9.2	[5.21, 16.1]	< 0.001
Private transport	2019	10.5	[9.10, 12.0]		1.75	[1.37, 2.24]		7.34	[6.19, 8.71]		8.27	[6.21, 11.0]		0.87	[0.63, 1.19]	
	2020	9.26	[5.4, 15.4]	0.66	2.57	[0.69, 9.52]	0.61	2.69	[2.05, 3.54]	< 0.001	3.52	[1.42, 8.75]	0.014	0.33	[0.12, 0.90]	0.013
Public transport	2019	13.5	[11.9, 15.2]		1.52	[1.29, 1.79]		31.2	[27.1, 36.1]		28.3	[23.4, 34.3]		3.81	[3.04, 4.78]	
	2020	14.1	[8.78, 21.8]	0.85	1.99	[0.90, 4.40]	0.54	12.4	[6.35, 24.1]	< 0.001	21.8	[9.27, 51.3]	0.49	3.07	[1.25, 7.54]	0.61

Values are means, aside from the “proportion visited” columns. Values in brackets are the 95% confidence intervals. All p-values are for coefficients for a year indicator variable in a bivariate regression of each column outcome. ^†^ Detailed information on other locations reported are provided in Additional file 1: Material S5

Our sensitivity analyses did not substantively change our conclusions (Additional file 1: Material S4). Estimated reductions in R_0_ between 2019 and 2020 R1 and 2020 R2 ranged from 40 to 50% and 38 to 48% respectively in the sensitivity analyses, compared to 42 and 45% respectively in the primary analysis. Excluding Fridays/Saturdays, contacts under 15 min, respondents with any missing contact age data or those aged 15–17 had no qualitative effect on age-mixing patterns. Adding children to the analysis highlighted the large number of contacts adults had with children pre-Covid, but did not affect the estimated reduction in R_0_ between 2019 and 2020. However, it was notable that adults’ close personal contact with children fell by less than almost any other age combination, looking similar to the pattern between 15 and 29 and 50–64 year olds.

## Discussion

We compared rates of close interaction contact and time spent with others indoors or on transport from surveys conducted in the same rural South African setting, both pre-Covid-19 in 2019 and during the first wave of cases in mid-2020. We found substantial declines in close contact numbers and in time spent at most indoor locations other than respondents’ own homes by 2020, suggesting that the combination of government NPIs and the ongoing epidemic substantially affected behaviour. Under the assumptions that close interaction contacts are the most important for infection transmission, and qualitatively the same in both years, we estimate a 42–45% reduction in the likely basic reproduction number between the two surveys. Given the substantial level of use of face coverings outside the home required and observed in South Africa [31], this reduction is likely to be an underestimate.

While it is difficult to disentangle the effects of NPIs and the state of the local epidemic, the fact that much of the decline between 2019 and 2020 was present by the first interview round – the majority of which occurred prior to the local mass arrival of Covid-19 – suggests that respondents in this area were complying with government mobility NPIs. By June and July these had been relaxed somewhat from the initial strict stay-at-home requirements, but entertainment and alcohol availability remained highly limited. The continuation of limited close interaction contact, even as NPIs were relaxed into August and beyond, highlights that the changes made in response to NPIs were maintained subsequently; it is hard to tell whether this was due to increased concern due to widespread local transmission or slow adjustment to changing policies.

Declines in close interaction contact were not homogenous. The age group with the greatest decreases in contact were those aged over 65, particularly for contact with others aged 50 and older, with declines of around three-quarters compared to pre-Covid. In contrast, the lowest declines were seen between those aged 15–29 and older adults—particularly those aged 30–49 years, i.e. one generation older. This pattern reflects the notably larger decline in non-household member contacts compared to household members in combination with the common presence of multiple adult generations within each household. This finding is similar that seen in other contact studies of rural Africans [32], and highlights the likely difficulty of protecting those most vulnerable to Covid-19 in settings such as this. A fuller understanding of the implications of these ongoing within-household intergenerational contacts will require focused qualitative work to determine whether young adults living in multigenerational households are able to maintain some social distance within houses, and how to potentially target messages to this population.

To consider the implications of behaviour change for an infection with aerosol transmission potential, we also measured time spent in indoor locations, and numbers of people present. Unsurprisingly, we found that time spent at home rose, while shared person-time spent in other homes dropped by 89% and at ‘other locations’ (largely school, work and shops) by 94%. This reduced potential for exposure in both public and private suggests that people are following rules both when they can and cannot be seen; these data are also consistent with other evidence from this area of reduced mobility in July and August compared even to earlier in 2020 [31]. Our respondents reported similar time spent attending health clinics in the two years, but reported that fewer others were present when they attended in 2020, perhaps reflecting improved social distancing policies within clinics. Overall, our indoor location data suggested nuanced decision-making by respondents during Covid-19, reducing less vital trips but maintaining necessary ones.

Even prior to Covid-19, the numbers of close interaction contacts reported in this area was substantially lower than that seen elsewhere in Africa [18, 32–35]. Low numbers of contacts may reflect the very large proportion of our respondents’ days spent at home – a mean of 19 h in 2019 and almost 22 h in 2020. This lack of mobility reflects very high local unemployment – under 25% of resident working-aged adults reported employment during the late-2019 demographic census. As a result, while household contact numbers are comparable to other African studies, contacts beyond the household appear to be substantially lower. In the context of a South African first wave of the Covid-19 epidemic that saw much smaller outbreaks in rural than urban areas, this lower baseline suggests a low rural R_0_ even before individuals increased their social distance.

## Strengths and limitations

There are limitations to this study. As with any observational study of human behaviour, care must be taken in generalising to other populations. While the pre-Covid-19 contact patterns we show here are consistent with those elsewhere in rural Africa [32], as are the changes seen with the arrival of Covid-19 [18], it is important to consider whether close contact patterns in rural lower-income settings may have different implications for disease prevention than patterns seen elsewhere.

In contrast to social contact surveys that used prospective diaries to capture information, we relied on recent recall – this may have led to some misreporting, but the delay was in all cases less than one week, limiting this concern. Our data were also self-reported rather than, for example, based on proximity detectors or mobility tracking. Self-report can lead to misreporting, although this effect is unlikely to have affected measures of change since we used the same approach for both years. Nevertheless, given the uncertainty of self-reports, absolute values of contact numbers should be treated with caution. It is also possible that respondents underreported more in 2020 insofar as they had visited places in contravention of the law and at the risk of fines or court appearances [36], if they believed that interviewers would report this or were ashamed of their behaviour. Self-report also has the benefit of providing richer data on the nature of each interaction. Conversely, we kept our questionnaires brief to minimize respondent fatigue, and as a result we do not have certain details about each contact, including whether a facemask was used during each interaction (although any such use of masks should have increased the drop in infectious potential from 2019 to 2020). We also do not have detailed information on how respondents changed their work, educational or other patterns of daily life; more in-depth interviews potentially using qualitative data collection methods would thus provide important additional insight into the effect of the epidemic and lockdowns on infection risk.

There are substantial strengths to this work. We were able to compare two studies with respondents drawn from the same well-defined sampling frame asking very similar questions about behaviour both shortly before and after the arrival of Covid-19 in the study area. The availability of longitudinal response data during the Covid-19 outbreak allowed us to observe behaviour changed as government NPIs and epidemic situations changed; although while patterns of behaviour over time can suggest effects, we cannot prove causality, something important if using our findings to design preventative interventions.

## Conclusion

In comparable surveys about social contacts conducted in the same rural South African location in 2019 and mid-2020, we find substantial declines in close physical and conversational contacts, and also in beyond-household sharing of indoor space. These findings suggest that the strict government NPIs implemented to mitigate the Covid-19 epidemic, in combination with the arrival of the epidemic itself in the local area, led to highly protective behaviours. It will be important to triangulate these findings with other information on the wider impact of such behaviour.

## Supplementary Information

**Additional file 1: Material S1. **Study instruments. **Material S2.** Lockdown levels and regulations. **Material S3**. Extended methods for statistical analysis. **Material S4.** Sensitivity analyses. **Material S5. **Detailed description of ‘other locations’ from non-close contact questions

## Data Availability

The datasets supporting the conclusions of this article are available through the AHRI data repository (https://data.ahri.org) in pseudonymized form  at 10.23664/ahri.uoandcsc.dataset.2021. Data can be requested directly from this repository and the corresponding author (Guy Harling) can be contacted to assist in this process.

## Abbreviations

AHRI: Africa Health Research Institute
COPD: Chronic Obstructive Pulmonary Disease
CSC: Covid Social Contact
NPI: Non-pharmaceutical interventions
R_0_: Reproduction number
REC: Research Ethics Committee
UKZN: University of KwaZulu-Natal
UO: Umoya omuhle
