# Supplementary material for: Impact of the Covid-19 epidemic and related social distancing regulations on social contact and SARS-CoV-2 transmission potential in rural South Africa: analysis of repeated cross-sectional surveys
Source: BMC Infect Dis. 2021 Sep 8;21:928. doi: 10.1186/s12879-021-06604-8 (PMC8424154; doi:10.1186/s12879-021-06604-8)
Supplement: Supplementary file 1 — Additional file 1: Material S1. Study instruments. Material S2. Lockdown levels and regulations. Material S3. Extended methods for statistical analysis. Material S4. Sensitivity analyses. Material S5. Detailed description of ‘other locations’ from non-close contact questions [file 12879_2021_6604_MOESM1_ESM.docx]

**Additional file**

**Title**: Impact of social distancing regulations and epidemic risk perception on social contact and SARS-CoV-2 transmission potential in rural South Africa: analysis of repeated cross-sectional surveys

**Additional file 1:** Material S1. Study instruments

|  | **Physical locations** |  |
| --- | --- | --- |
|  | Make a list of all the indoor places you used on [day] between 12am and 12am*. The interviewer will make a list of all of the places on a piece of paper. This will only be used to help you remember the places. Do not include locations that you did not go inside (e.g. shops where you were served through a hatch). Include tents that were enclosed, but not tents that only had a roof. This should include your own home if you spent time there. If you visited a location more than once, then list it more than once, and answer the subsequent questions for each time you visited. For workplaces, put the type of workplace (e.g. shop). |  |
| ph01 | How many places are on your list of buildings visited? | Integer |
|  | [Loop over list from PH01 for PH02-PH07] |  |
| ph02 | What type of location was it? | 0 "Own home" 1 "Other house on plot" 2 "House off plot" 3 "Church" 4 "Clinic" 5 "Community building" 6 "Creche" 7 "Gym" 8 "Library" 9 "Mall / Shops" 10 "in Bar/ Nightclub" 11 "Spaza" 12 "School" 13 "Salon/Barber" 14 "In a tent" 15 "Counselling Centre" 16 "Guest House" 17 "Office" 18 "Workshop" 19 "Restaurant" 96 "Other" 98 "Prefer not to say" |
| ph03^†^ | Which isigodi (local area) was it in? | [List of izigodi (local areas)] |
| ph04^†^ | Is this place your workplace? | 0 “No” 1 “Yes” 99 “Don’t know 98 "Prefer not to say" |
| ph05 | What time did you arrive? | Time |
| ph06 | How long did you spend there? | time |
| ph07 | How many people (adults and children) were there, halfway through the time you were there? | integer |
| ph08 | How many of those people were children aged < 15? | Integer |
| ph09 | On how many days did you visit this location in the past week? | integer |
|  | Make a list of all the transport you used on [day] between 12am and 12am*. If you used a method of transport more than once (for instance a trip on two different taxis), then list it more than once, and answer the subsequent questions for each time you used it. |  |
| ph10 | How many trips are on your list of trips made? | integer |
|  | [Loop over list from PH8 for PH9-13] |  |
| ph11 | What type of transport was it? | 0 "Taxi" 1 "Bakkie" 2 "Private car" 3 "Bus" 4 “Motorbike” 5 “Bicycle” 96 "Other" 98 "Prefer not to answer" |
| ph12^†^ | Where did it start? | [List of izigodi (local areas)] |
| ph13^†^ | Where did it end? | [List of izigodi (local areas)] |
| ph14 | What time did it start? | time |
| ph15 | How long did the journey take? (Don't worry if approximate) | time |
| ph16 | How many people (adults and children) were on the vehicle at the start of your trip? | integer |
| ph17 | How many of those people were children aged < 15? | integer |
| ph18^†^ | Is this the transport you typically use on [day]? |  |
| ph19^†^ | [If PH18 == 0] Do you usually make no trips using transport on [day]s? | 0 “No” 1 “Yes” 99 “Don’t know 98 "Prefer not to say" |
|  | **Social contacts** **2019** |  |
| sc01 | Make a list of all the people you spoke to or touched on [day]. Make a note of which are in your household. Include face-to-face meetings only (phone calls don't count). By [day], we mean between 12am and 12am.  The interviewer will make a list of all of the people on a piece of paper. You can give us nicknames or their relationship to you if you prefer (for example, 'my mother'). |  |
|  | [Loop questions SC02-SC09 for a random 10 people from SC01, or all if fewer than 10 reported] |  |
| sc02 | How many people are on your list? | integer |
| sc03 | How many of those people are members of your household? | integer |
| sc04 | What is your relationship to this person? | 0 “You live together” 1 “Neighbour” 2 “Relative” 3 “Work colleague” 4 “Friend” 5 “Stranger” 96 “Other” 98, “Prefer not to answer” |
| sc05 | How old do you think they are? | integer |
| sc06 | Are they male or female? | 1 “Male” 2 “Female” 99 “Don’t know” 98 “Prefer not to answer” |
| sc07 | How much time did you spend with them in total? | time |
| sc08 | Did you touch them, for instance with a handshake, a hug or a kiss? | 0 “No” 1 “Yes” 99 “Don’t know 98 "Prefer not to say" |
| sc09 | How often do you typically see them? | 0 “6 or 7 days a week” 1 “2-5 days a week” 2 “Once a week” 3 ”Once a week to once a month” 4 “Less than once a month” 5 “Never before” 98 “Prefer not to answer” |
|  | **Social contacts** **2020** |  |
| sc01 | We will now ask you to remember who you have been in contact with yesterday, between 5am yesterday and 5am today. We are only interested in direct contacts, which are people who you met in person and with whom you exchanged at least a few words, or with whom you had physical contact (e.g. a handshake, embracing, kissing, contact sports). Note that if you only spoke to someone over the phone or internet, they should not be included.  Please write the nickname of each other person in your household. Note that this nickname is only needed to make it easier for you to complete the survey, so please pick a nickname that will help you identify each household member later in the questionnaire. Nicknames are not visible to anyone outside of this survey. |  |
|  | [Loop over list from SC01 for SC02] |  |
| sc02 | Which of these household members did you have direct contact with in person, between 5am yesterday and 5am today? | Yes, No |
| sc03 | And what other people did you have direct contact with in person, between 5am yesterday and 5am today? Please think about anyone else you had direct contact with. This could include friends, family, work colleagues, or people you spoke to in shops and so on. The order in which you give these names does not matter. However, it is easiest to give them in chronological order, e.g. when I woke up, I saw Busi and Thabo at breakfast. I then drove to my work, where I met with Thandiwe and Sfiso. On my way back home, I stopped at a petrol station, where I had a brief chat with the shop assistant. Etc.  Please write the nickname of each person you had direct contact with. Note that this nickname is only needed to make it easier for you to complete the survey, so please pick a nickname that will help you identify each contact later in the questionnaire. Nicknames are not visible to anyone outside of this survey. |  |
|  | [Loop over list from SC03 for SC04-SC11] |  |
| sc04 | What age is NAME? Please give an estimate if you are not sure. | 0-4, 5-9, 10-14, 70-74, 75-79, 80-84, 85+ |
| sc05 | What gender is NAME? | 0 Male; 1 Female; 96 Other; 98 Prefer not to answer; 99 Don’t know |
| sc06 | What is NAME’s relationship to you? | 0 They are a family member who is not in my household; 1 They are someone I work with; 2 They are someone I go to school, college or university with; 3 They are a friend; 96 Other; 98 Prefer not to answer |
| sc07 | Before the coronavirus epidemic started, how often did you usually have direct contact with NAME? A direct contact is when you meet with this person in person and when you exchange at least a few words, or when you have physical contact (e.g. handshake, embracing, kissing, contact sports). Please do not include times that you speak to them over the phone or internet. | 0 Every day or almost every day; 1 About once or twice a week; 2 Every 2-3 weeks; 3 About once per month; 4 Less often than once per month; 5 Never met them before; 98 Prefer not to answer |
| sc08 | When you had direct contact with NAME yesterday, did you have | 0 Physical contact (any sort of skin-to-skin contact such as e.g. hand shaking, embracing or kissing); 1 Non-physical contact (you did not touch the person); 98 Prefer not to answer |
| sc09 | Where did you have direct contact with NAME? [Do not read out list] | 0 "Own home" 1 "Other house on plot" 2 "House off plot" 3 "Church" 4 "Clinic" 5 "Community building" 6 "Creche" 7 "Gym" 8 "Library" 9 "Mall / Shops" 10 "in Bar / Spotini / Nightclub" 11 "Spaza" 12 "School" 13 "Salon/Barber" 14 "In a tent" 15 "Counselling Centre" 16 "Guest House" 17 "Office" 18 "Workshop" 19 "Restaurant" 96 "Other" 98 "Prefer not to say" |
| sc10 | Please estimate the total amount of time you spent with [NAME] in person yesterday | time |
| sc11 | Was the time you spent with [NAME] yesterday inside or outside? [Select all that apply] | 0 Inside; 1 Outside; 2 Both |

The questions listed here are the relevant sections of the 2019 Umoya Omuhle survey and the 2020 Covid Social Contacts survey, and not the complete questionnaires in each case. * In 2020 the times were 5am and 5am. ^†^ Questions only asked in 2019.

Additional file 1: Material S2. Lockdown levels and regulations

| **Lockdown level** | **Date started** | **Indicative regulation changes** |
| --- | --- | --- |
| 5 | 26-Mar-20 | Complete alcohol sale ban; complete tobacco sale ban; no movement outside home without permit except to buy essential goods or seek medical care; substantial national border closure; gatherings banned except limited funerals; facemasks mandatory in public. |
| 4 | 01-May-20 | Exercise allowed within 5km of residence between 0600-0900; curfew 2000-0500 except with permit; limited public transport allowed; inter-municipality travel remained largely banned. |
| 3 | 01-Jun-20 | Curfew shortened to 2100-0400; small group exercise allowed between 0600-1800; intra-provincial travel allowed; limited religious gatherings & non-contact professional sports allowed; non-entertainment/leisure business can reopen, including restaurants. |
| 2 | 17-Aug-20 | Inter-provincial travel allowed; rented accommodation allowed; alcohol (limited hours) & tobacco sales allowed; social visiting allowed; fitness centres reopen. |
| 1 | 21-Sep-20 | International travel allowed; increased capacity at gatherings; relaxation of alcohol sale regulations |

Additional file 1: Material 2. Extended methods for statistical analysis

***Weighting***

To ensure both comparability across surveys and meaningfulness of results we weighted all our observed data to match the demographic surveillance area census population from which respondents were drawn. Additionally, for the UO data, when using contact characteristics, for respondents naming more than 10 contacts, we resampled from the 10 random contacts upon which more detailed information was provided, up to the total number of reported contacts. For the 2020 CSC data, we weighted respondents to account for stratified sampling and non-response by age, sex, comorbidity status, to match the Vuk’uzazi respondent sample. We then further weighted by age and sex to account for Vuk’uzazi non-response, to reach the census distribution. The UO data was weighted to the same age and sex distribution. As no respondents aged 15-17 were interviewed in the UO survey, respondents aged 18-29 were assumed to be representative of 15-29 year olds in the main analysis. Finally, we weighted responses by day of week, so that weekdays accounted for 5/7 of all observations, and weekends the remainder.

Age-mixing matrices were made symmetrical by setting the estimated rate of contact between each person in age group *i* and age group *j* equal to mean of the rates reported by respondents in each age group, using the age distribution of the census population to calculate the rates.

***Management of incomplete data***

Some respondents reported not knowing some or all of their contacts’ ages. We excluded from all the age-mixing matrices and R_0_ estimates any respondent who gave this response for all their close contacts in a round (n=2 in UO, n=10 in CSC R1, n=6 in CSC R2). People with missing data for all of their close contacts had a slightly lower mean number of close contacts (mean of 1.5 vs 7.3 in UO,mean of 3.5 vs 4.0 in CSC R1, mean of 3.2 vs 4.0 in CSC R2; all unweighted); this may reflect them being less engaged with the survey or a genuinely lower number of contacts. For respondents giving only some ages, we upweighted the known-aged contacts for the respondents to replace missing-age ones in the ‘best estimate’ age-mixing matrices, and excluded missing-age contacts from the resampling process when estimating confidence intervals for the age mixing matrices and when estimating the relative R_0_ reduction (while keeping the total number of contacts to sample for each respondent equal to the total number of contacts reported).

For the transport and location analysis, we excluded respondents reporting no location in the past 24-hours (including their own home) as implausible (n=1 in UO; n=1 R1, n=6 R2 in CSC). We used the same weights as for the close contact analyses.

***Sensitivity analyses***

For the analysis incorporating children: Since we ignored contacts aged under 15 in our primary analysis, because they were not eligible for either survey, we incorporated children into our analysis using the data on contacts with children reported by adults to calculate contact rates between children and adults. As we had no data on contact rates between children, we used data from a previous social contact study in South Africa to estimate these, making the assumption that the ratio of contact rates between 0-14 year old and contact rates between 15-29 year olds was the same (Johnstone-Robertson, Mark et al. 2011). In estimating R0, we also assumed that the probability of infection per contact was 44% lower for children than for adults (Viner, Mytton et al. 2020).

For the analysis excluding 15-17 year olds: As exact contact ages were not collected in CSC, we assumed that the proportion of all contacts aged 15-19 who were aged 18-19 was proportional to the proportion of the census population aged 15-19 who were aged 18-19.

**References**

Johnstone-Robertson SP, Mark D, Morrow C, Middelkoop K, Chiswell M, Aquino LD, Bekker L-G, Wood R. Social mixing patterns within a South African township community: implications for respiratory disease transmission and control. *American Journal of Epidemiology* 2011; 174(11): 1246-55.

Viner, RM, Mytton OT, Bonell C, Melendez-Torres G, Ward H, Hudson L, Waddington C, Thomas J, Russell S, Van Der Klis F. Susceptibility to SARS-CoV-2 infection among children and adolescents compared with adults: A systematic review and meta-analysis. *JAMA Pediatrics* 2020. doi: 10.1001/jamapediatrics.2020.4573.

Additional file 1: Material S3. Sensitivity analyses

**Estimated percent reductions in R0**

|  | **2019 to 2020 R1** | |  | **2019 to 2020 R2** | |  | **2020 R1 to 2020 R2** | | |
| --- | --- | --- | --- | --- | --- | --- | --- | --- | --- |
|  | **Mean (95% PR)** | **p-value** |  | **Mean (95% PR)** | **p-value** |  | **Mean (95% PR)** | **p-value** |  |
| Primary estimate | 41.7% (13.6, 59.1%) | 0.004 |  | 45.1% (24.2, 60.8%) | <0.001 |  | 2.3% (-53.0, 43.5%) | 0.4 |  |
| 1. Day of week weights | 42.1% (15.1, 59.1%) | 0.002 |  | 47.6% (28.9, 61.8%) | <0.001 |  | 6.4% (-43.6, 44.4%) | 0.4 |  |
| 2. Exclude respondents with missing contact ages | 49.5% (37.9, 59.6%) | <0.001 |  | 45.0% (22.0, 61.5%) | <0.001 |  | -10.2%, (-63.4%, 27.6%) | 0.5 |  |
| 3. Exclude short duration contacts | 43.9% (14.3, 62.2%) | 0.004 |  | 46.3% (24.3, 62.8%) | <0.001 |  | -0.015 (-62.1, 45.0%) | 0.5 |  |
| 4. Include children | 40.1% (18.4, 54.0%) | <0.001 |  | 41.4% (23.7, 55.3%) | <0.001 |  | 0.007 (-42.6, 34.5%) | 0.5 |  |
| 5. Exclude 15-17 year olds | 46.6% (34.0, 51.0%) | <0.001 |  | 37.8% (6.3, 59.2%) | 0.001 |  | -17.8% (-84.9, 27.1%) | 0.5 |  |
| 6. June-August only in 2019 data | 41.1% (12.8, 58.8%) | 0.005 |  | 44.5% (23.3, 60.7%) | <0.001 |  |  |  |  |
| 7. Urban/rural weights | 42.0% (14.2, 59.3%) | 0.004 |  | 45.4% (24.6, 61.0%) | <0.001 |  |  |  |  |

**1. Day of week weights**

**
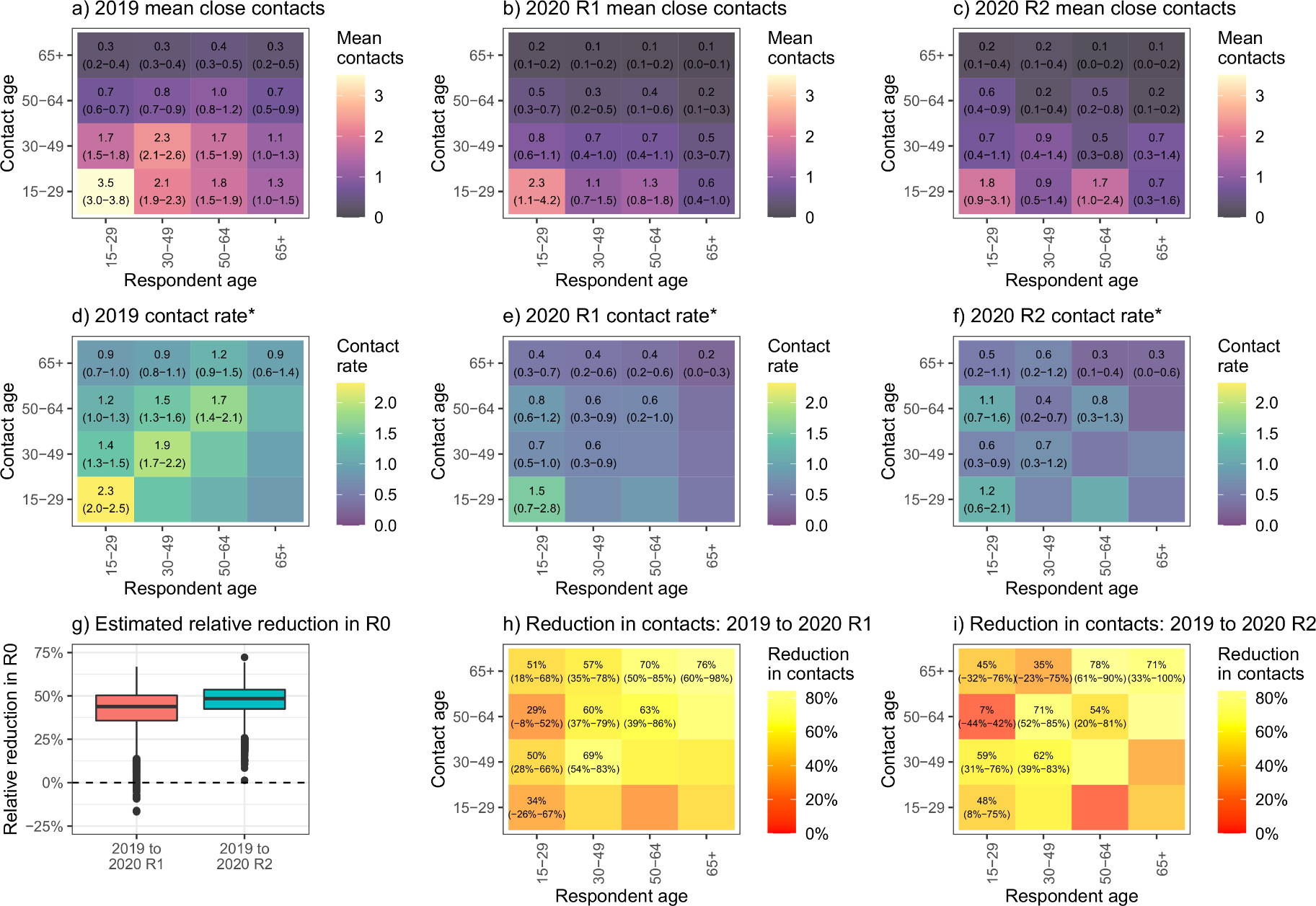
**

**2.Exclude respondents with missing contact ages**

**
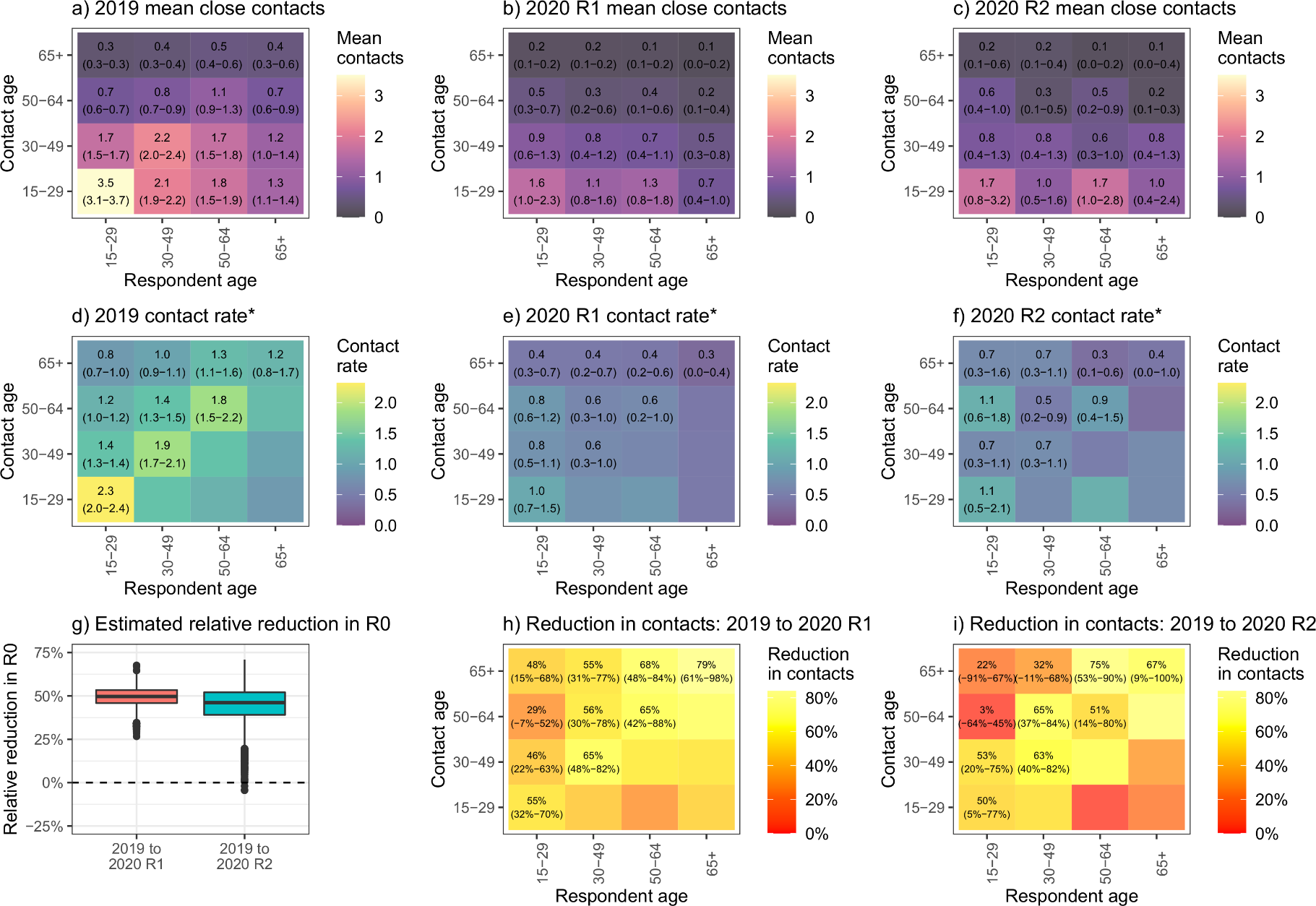
**

**3. Exclude short duration contacts**

**
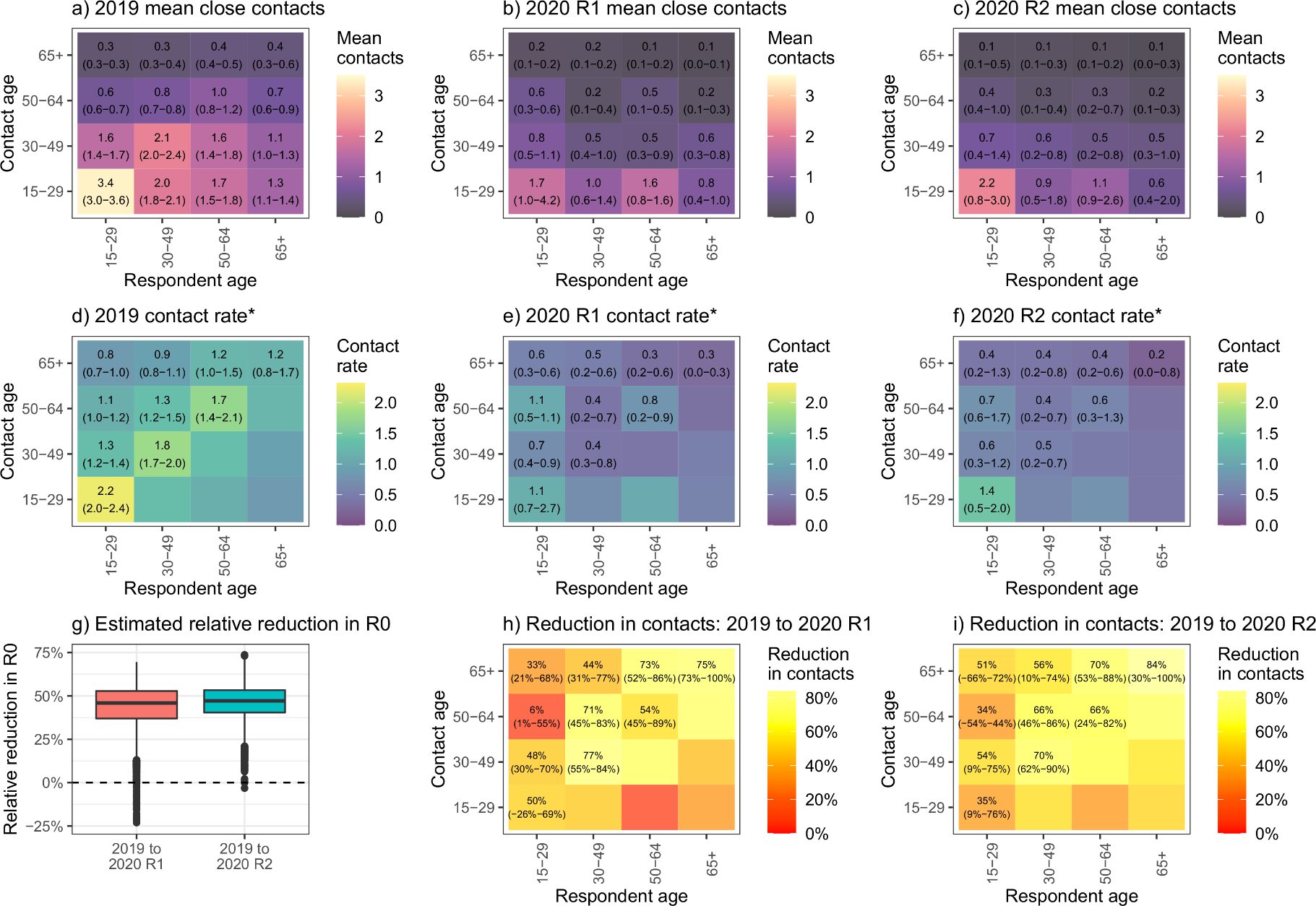
**

**4. Include children**

**
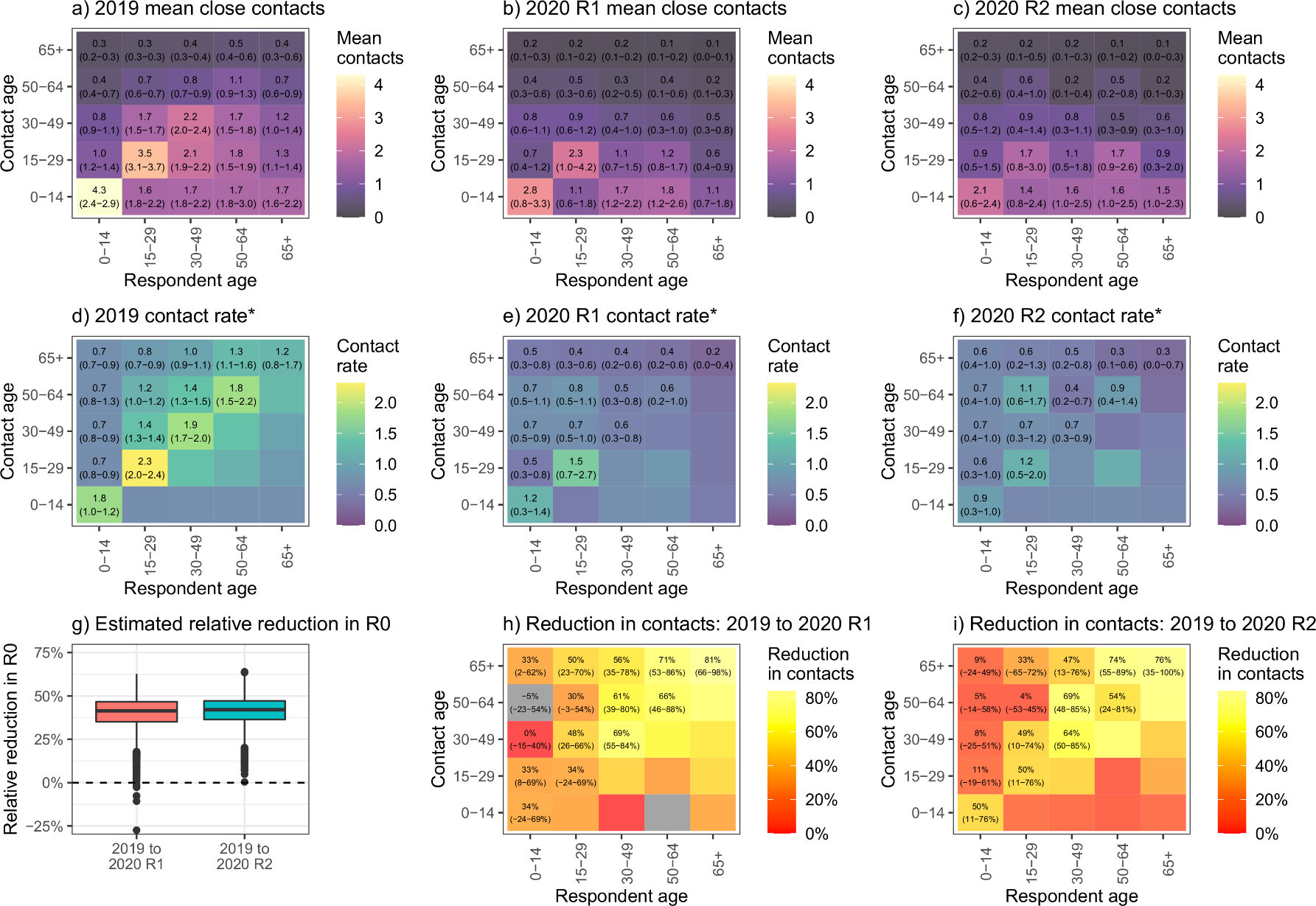
*** The colour scales for a)-c) of this graph are different than those used in the other figures

**5. Exclude 15-17 year olds**


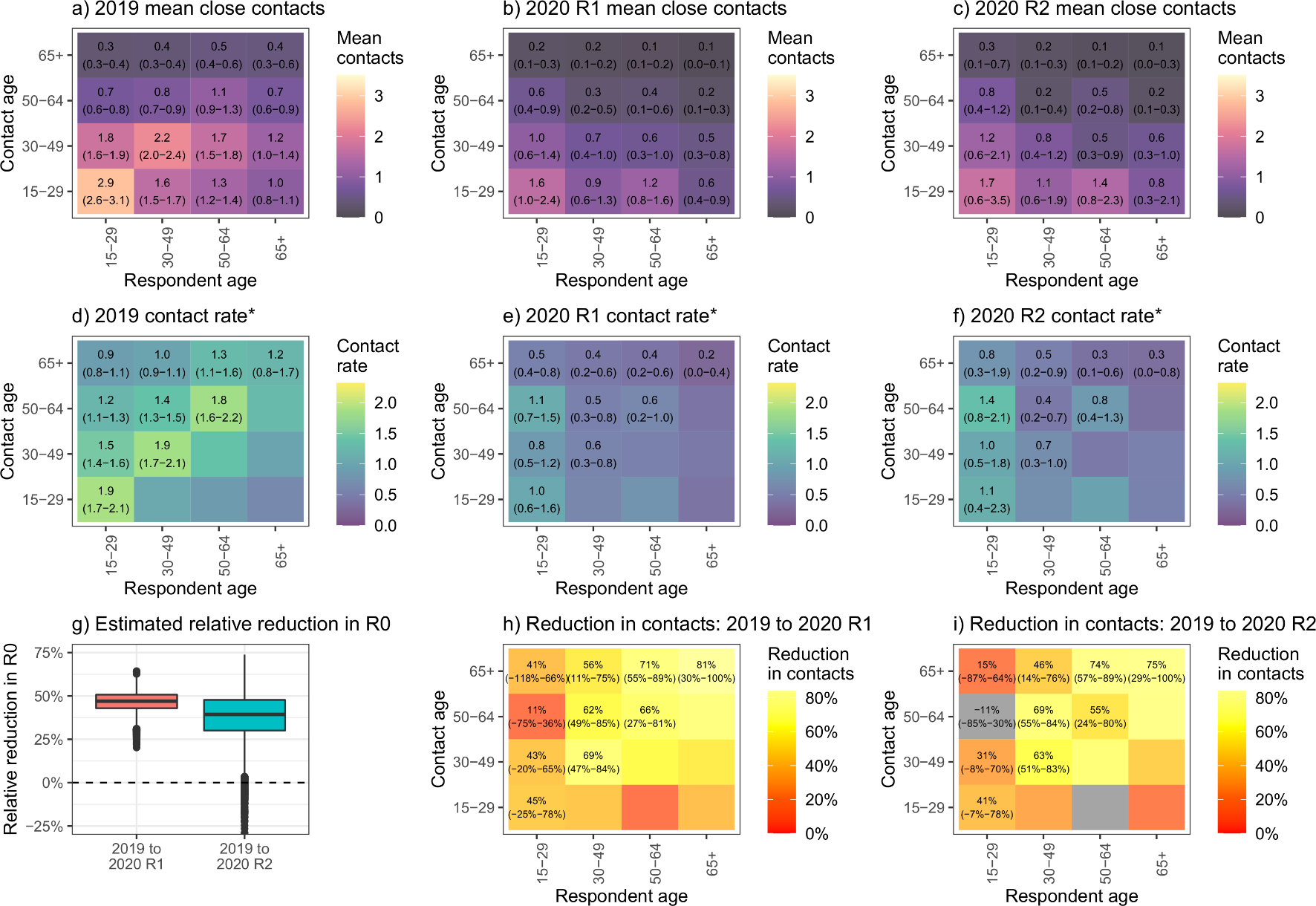

* Grey in graph i) indicates an increase in contact numbers and rates

**6. Only use UO data from June-August 2019**


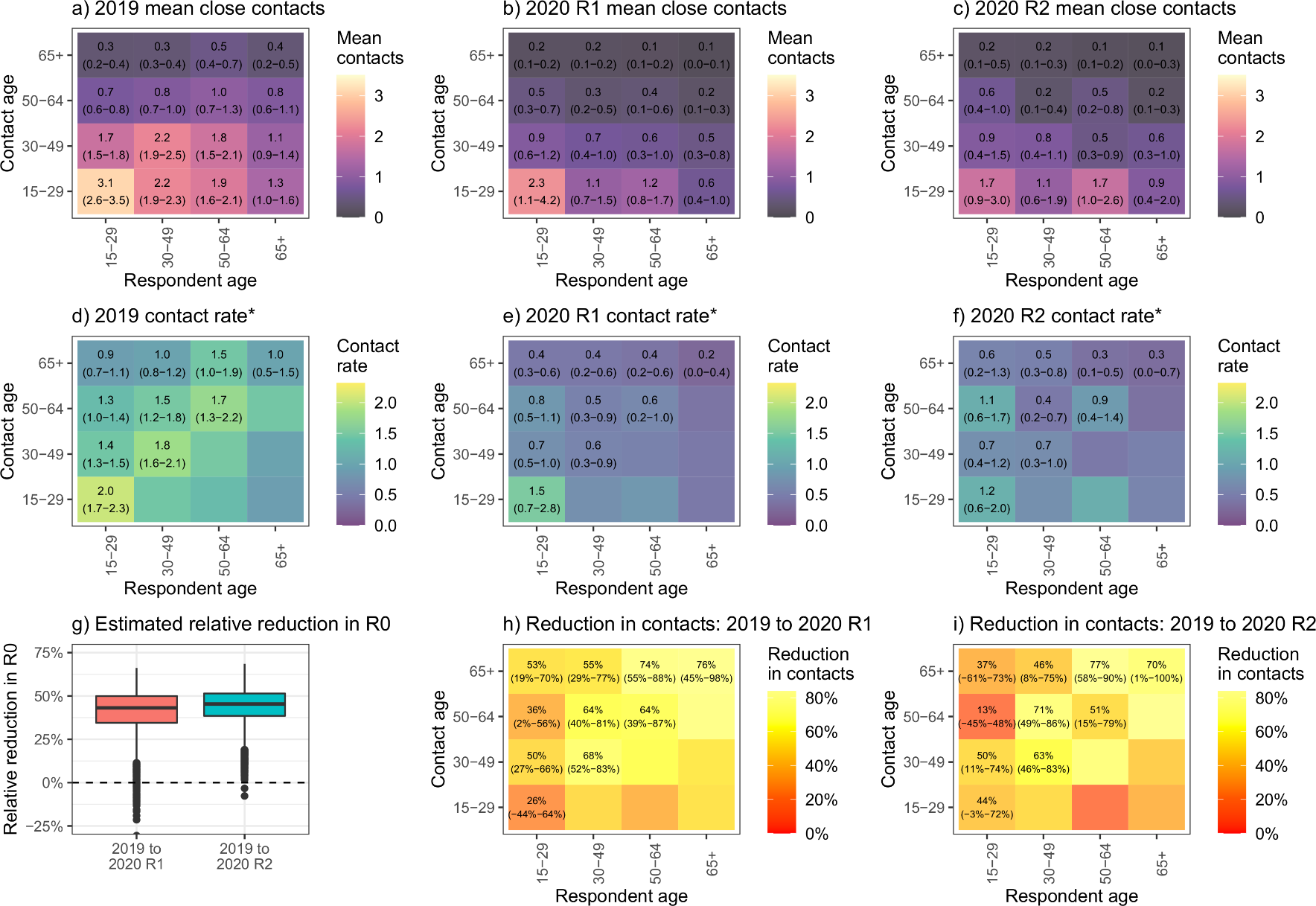


**7. Weight the 2019 data to the proportion urban/peri-urban and rural in the census**


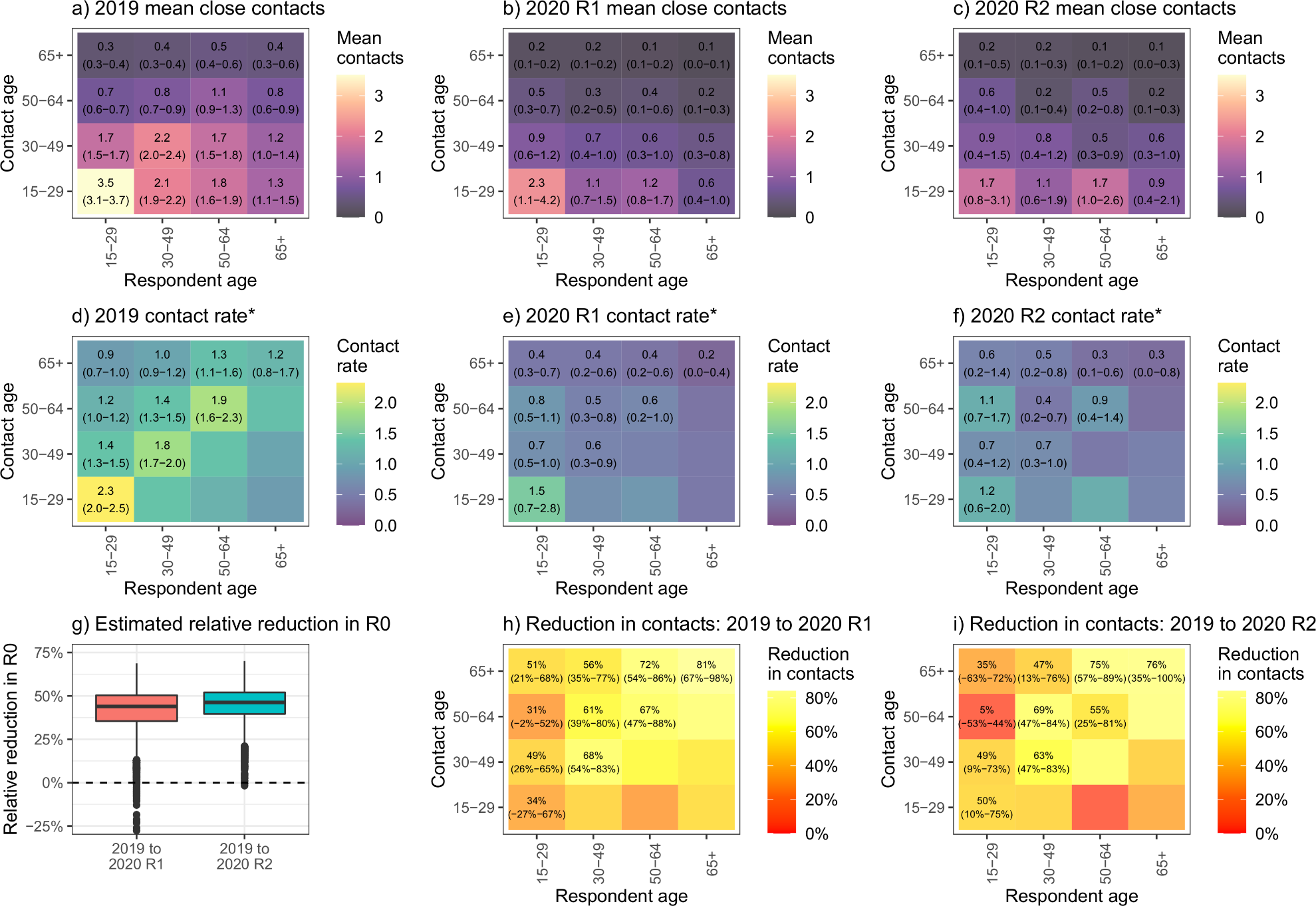


Additional file 1: Material S4. Detailed description of ‘other locations’ from non-close contact questions

| **Location** |  | **Proportion**  **visited (%)** | | **p** |  | **Hours**  **if visited** | | **p** |  | **People**  **per visit** | | **p** |  | **Contact-hours**  **if visited** | | **p** |  | **Contact-hours** | | **p** |
| --- | --- | --- | --- | --- | --- | --- | --- | --- | --- | --- | --- | --- | --- | --- | --- | --- | --- | --- | --- | --- |
|  |  |  |  |  |  |  |  |  |  |  |  |  |  |  |  |  |  |  |  |  |
| Workshop/office | 2019 | 8.0 | (6.8, 9.4) |  |  | 8.4 | (7.8, 9) |  |  | 39 | (20, 76) |  |  | 363 | (178, 743) |  |  | 29 | (14, 60) |  |
|  | 2020 | 3.0 | (1.1, 7.6) | 0.041 |  | 8.4 | (7.3, 9.8) | 0.94 |  | 3.6 | (2.8, 4.7) | 0.0076 |  | 27 | (17, 41) | 0.012 |  | 0.8 | (0.3, 2.4) | 0.0089 |
|  |  |  |  |  |  |  |  |  |  |  |  |  |  |  |  |  |  |  |  |  |
| Mall/shop/salon | 2019 | 8.3 | (7.1, 9.8) |  |  | 4.0 | (3.5, 4.6) |  |  | 168 | (129, 219) |  |  | 702 | (498, 988) |  |  | 59 | (40, 85) |  |
|  | 2020 | 12.0 | (6.9, 20) | 0.22 |  | 3.0 | (1.6, 5.7) | 0.28 |  | 23 | (8.7, 62) | <0.001 |  | 44 | (21, 94) | <0.001 |  | 5.3 | (2.3, 12) | <0.001 |
|  |  |  |  |  |  |  |  |  |  |  |  |  |  |  |  |  |  |  |  |  |
| School/crèche | 2019 | 6.8 | (5.7, 8.2) |  |  | 6.5 | (5.9, 7.1) |  |  | 84 | (53, 135) |  |  | 543 | (331, 891) |  |  | 37 | (22, 62) |  |
|  | 2020 | 3.0 | (1, 8.8) | 0.14 |  | 5.5 | (3.4, 9.1) | 0.32 |  | 15 | (4.4, 50) | 0.0012 |  | 62 | (27, 143) | <0.001 |  | 1.9 | (0.6, 6) | <0.001 |
|  |  |  |  |  |  |  |  |  |  |  |  |  |  |  |  |  |  |  |  |  |
| Church | 2019 | 4.1 | (3.2, 5.2) |  |  | 3.9 | (3.3, 4.6) |  |  | 54 | (42, 69) |  |  | 220 | (165, 293) |  |  | 9.0 | (6.3, 13) |  |
|  | 2020 | 0.8 | (0.3, 2.4) | 0.0040 |  | 6.6 | (0.5, 84.4) | 0.56 |  | 5.8 | (2.8, 12) | <0.001 |  | 34 | (3.1, 376) | <0.001 |  | 0.3 | (0.1, 1.4) | <0.001 |
|  |  |  |  |  |  |  |  |  |  |  |  |  |  |  |  |  |  |  |  |  |
| Bar/restaurant | 2019 | 2.5 | (1.8, 3.4) |  |  | 4.3 | (3.2, 5.7) |  |  | 60 | (29, 126) |  |  | 178 | (105, 301) |  |  | 4.5 | (2.5, 8) |  |
|  | 2020 | 0.0 |  |  |  |  |  |  |  |  |  |  |  |  |  |  |  |  |  |  |
|  |  |  |  |  |  |  |  |  |  |  |  |  |  |  |  |  |  |  |  |  |
| Other*/unknown | 2019 | 4.8 | (3.9, 5.9) |  |  | 5.4 | (4.5, 6.4) |  |  | 97 | (58, 163) |  |  | 441 | (258, 754) |  |  | 21 | (12, 37) |  |
|  | 2020 | 6.2 | (3.2, 11.7) | 0.47 |  | 7.9 | (3.6, 17.3) | 0.38 |  | 6.6 | (2.9, 15) | <0.001 |  | 18 | (7.4, 43) | <0.001 |  | 0.9 | (0.4, 2.4) | <0.001 |

Values are means, aside from the “proportion visited” columns, and 95% confidence intervals in parentheses. All p-values are for coefficients for a year indicator variable in a bivariate regression of each column outcome. *Other includes libraries, gyms, community buildings, and guest houses.
